# An In Vitro Study on the Effects of Hydrogen Peroxide-Based Bleaching Agents on Enamel: Field Emission Scanning Electron Microscopy (FESEM) With Energy Dispersive Spectroscopy (EDS) Evaluation

**DOI:** 10.7759/cureus.58318

**Published:** 2024-04-15

**Authors:** Sreelakshmi PS, Chellaswamy Savrimalai Karumaran, Arjun R S, Corrine Esther Manuel, Jevina Christy V, Roselin Stalin

**Affiliations:** 1 Conservative Dentistry and Endodontics, Chettinad Dental College and Research Institute, Chennai, IND; 2 Conservative Dentistry and Endodontics, Ragas Dental College and Hospital, Chennai, IND; 3 Conservative Dentistry and Endodontics, Jaya Prakash (J P) Dental Specialists, Thiruvananthapuram, IND; 4 Conservative Dentistry and Endodontics, Sudhagar Dental Clinic, Vellore, IND; 5 Conservative Dentistry and Endodontics, Rajas Dental College and Hospital, Tirunelveli, IND; 6 Conservative Dentistry and Endodontics, Thai Moogambigai Dental College and Hospital, Chennai, IND

**Keywords:** energy dispersive x-ray analysis (edax), eds, demineralization, fesem, hydrogen peroxide, bleaching agents

## Abstract

Aim and objective

The aim of the present in vitro study is to evaluate the morphological and elemental alterations in enamel following bleaching with hydrogen peroxide-based bleaching agents of different concentrations and pH values when exposed to different treatment times.

Materials and method

Twenty extracted maxillary central incisors were selected for the study. Tooth samples were prepared by sectioning the tooth cervico-incisally into two halves. The teeth were divided into different groups based on the bleaching protocol and bleaching agent applied: Group IA, Group IB, Group IIA, and Group IIB. Group IA received a 35% hydrogen peroxide-based bleaching agent of pH 6 for 10 minutes with light application. Group IB received a 35% hydrogen peroxide-based bleaching agent of pH 6 for 30 minutes with light activation. Group IIA received a 40% hydrogen peroxide-based bleaching agent of pH 8.5 for 10 minutes with chemical activation. Group IIB received a 40% hydrogen peroxide-based bleaching agent of pH 8.5 for 30 minutes with chemical activation. The morphology of the enamel before and after the application of the bleaching agent was evaluated using field emission scanning electron microscopy. The elemental analysis of enamel between the control and test samples was done with the help of energy dispersive spectroscopy.

Results

Paired t-test was used to analyze the data obtained from the study. The test samples showed erosive alterations in enamel surface morphology and also a decrease in the concentration of minerals when compared to the corresponding control groups.

Conclusions

The present study evidences the erosive potential of hydrogen peroxide-based bleaching agents. It can be concluded that bleaching agents containing high concentrations of hydrogen peroxide with acidic pH can cause mineral loss and surface erosion of enamel which is extremely detrimental to the tooth integrity.

## Introduction

A beautiful smile is an indication of youth, health, vitality, and success [1]. The general perception among the public is that the lighter the tooth color more attractive the smile [2]. Hence whitening or bleaching has gained tremendous popularity in recent years. It is regarded as the most conservative treatment modality in achieving brighter smiles when compared to the other available restorative procedures [2]. Commonly used bleaching agents include hydrogen peroxide (HP), carbamide peroxide (CP), sodium perborate, peroxymonosulphate, peroxide plus metal catalysts, and oxidoreductase enzymes [3,4]. These peroxides are strong oxidizing agents that dissociate to form free radicals, reactive oxygen species, and hydrogen peroxide anions that diffuse through enamel and dentin to decompose the intrinsic chromophores into smaller molecules [1]. Despite the numerous advantages of tooth bleaching, this interaction between the peroxide and dental hard tissues is thought to cause ultrastructural alterations in enamel [5]. The two important factors that contribute to the alterations in enamel are the pH and concentration of the bleaching agent [6]. Most of the bleaching agents are commercially available at a lower pH to stabilize the active ingredients and facilitate bleaching [7]. This acidic pH of the bleaching agent causes erosive alterations in the enamel topography, making the safety of its application in dentistry questionable [8]. Studies have also evidenced a reduction in the level of minerals like calcium, phosphorus, sulfur, potassium, and organic components in dental tissues after bleaching [9]. Several studies were conducted in the past to evaluate the mineral and ultrastructural changes in enamel, but most of them were proved inconclusive. Among the various methods used to assess the demineralization of enamel, field emission scanning electron microscopy (FESEM) with energy dispersive spectroscopy (EDS) has proven to be very effective [10]. The present study aimed to quantitatively determine the erosive potential of commonly used bleaching agents and analyze the alteration in the concentration of various elements present in enamel like calcium, phosphorous, magnesium, potassium, sodium, and chlorine present following bleaching. An understanding of the alterations in the mineral content throws light on the demineralization and remineralization processes taking place in enamel after bleaching. A thorough knowledge of the morphological changes, amount of mineral loss, and distribution of minerals on the eroded enamel surface is essential to overcome the side effects of the bleaching procedure.

## Materials and methods

**Figure 1:**
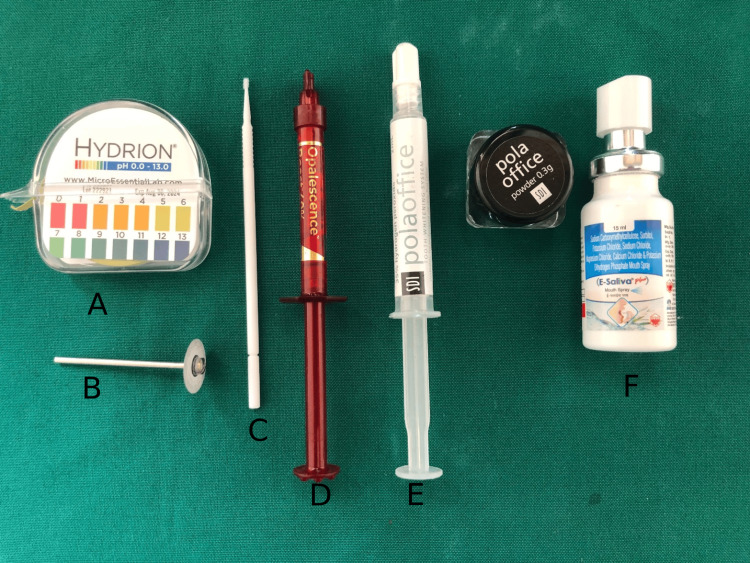
Armamentarium used for the study A: Hydrion pH paper, used to test the pH of the bleaching agents. B: Dentgallop diamond disc, used to section maxillary central incisors. C: Applicator tip, used to apply bleaching agent on the test sample. D: Opalescence Boost 40% bleaching agent. E: Pola office 35% bleaching agent. F: Artificial saliva, used to store the samples during the course of the experiment.

Twenty extracted human maxillary central incisors were collected from the Department of Oral and Maxillofacial Surgery, Ragas Dental College and Hospital, Chennai, India. Maxillary central incisors free of any enamel defects, restorations, caries, mineral loss, or cracks were selected for the study. The armamentarium used in the study is depicted in Figures 1A-1E.

**Figure 2:**
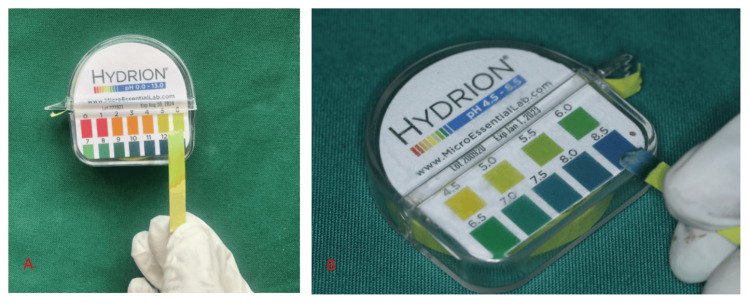
Measuring the pH A: Measuring the pH of Pola Office 35% with Hydrion pH paper. B: Measuring the pH of Opalescence Boost 40% with Hydrion pH paper.

The teeth were thoroughly washed under running water, and the roots were debrided of the periodontal tissues and debris. The teeth samples were stored in artificial saliva during the intermittent period to mimic intraoral conditions and to utilize its remineralizing benefits. Commercial bleaching agents namely Pola Office 35% and Opalescence Boost 40% were utilized in the study. The pH of both the bleaching agents was measured using Hydrion pH paper with a color chart. The pH of 35% Pola Office and Opalescence Boost 40% was found to be neutral (6) and alkaline (8.5), respectively (Figures 2A, 2B).

**Figure 3:**
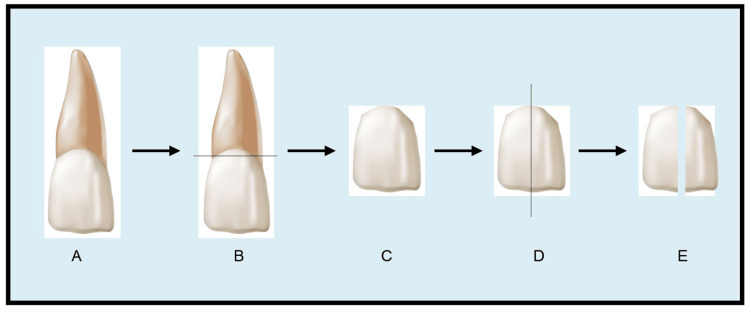
Diagrammatic illustration of tooth sample preparation A: Represents human maxillary central incisor selected for the study. B: Represents sectioning of the tooth mesiodistally 2 mm below the cementoenamel junction. C: Represents the tooth crown obtained after discarding the sectioned root portion. D: Represents sectioning of tooth crown cervico-incisally to obtain two longitudinal sections of the crown. The crown is sectioned in this fashion to obtain a control and test sample from the same tooth. E: Represents the two longitudinal sections obtained after sectioning the crown. One section serves as the control, while the other section serves as the test. The image is created using Adobe Illustrator and Adobe Photoshop.

The selected teeth were randomly divided into two groups: Group I and Group II, depending on the concentration of bleaching agent they received. Each group was further divided into subgroups depending on the time for which the bleaching agent was applied on the enamel surface. Group I was further divided into subgroups: Group IA and Group IB. Group II was subdivided into Group IIA and Group IIB. Each subgroup included five teeth. To prepare the specimens for the study, the tooth was cut 2 mm below the cementoenamel junction (CEJ) using a diamond disc (Dentgallop diamond disc). To obtain control and test samples, the same tooth was further cut longitudinally along its long axis (Figures 3A-3E).

**Figure 4:**
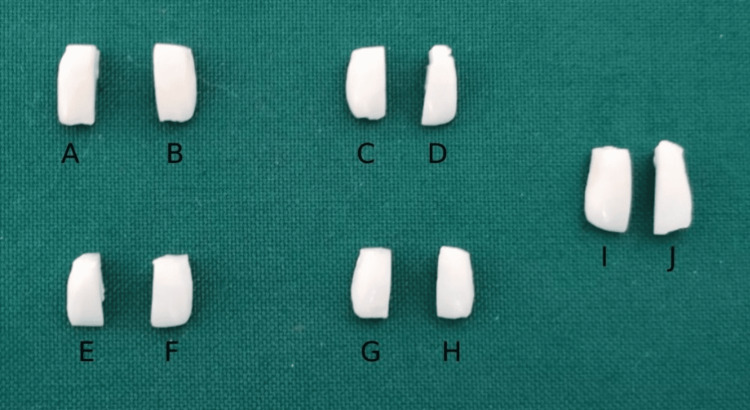
Image represents control and test samples of Group IA prepared by sectioning the maxillary central incisors A, C, E, G, I: Represent the control samples of Group IA obtained by sectioning maxillary central incisors. B, D, F, H, J: Represent the test samples of Group IA obtained by sectioning maxillary central incisors.

Forty samples were obtained in the similar manner from the selected 20 maxillary central incisors. One half of the tooth served as the control, and the other half of the same tooth served as the test sample (Figures 4A-4J).

**Figure 5:**
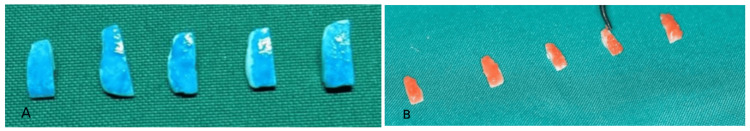
Labial aspect of the test samples A: Pola Office 35% applied on the labial aspect of the test samples. B: Opalescence Boost 40% applied on the labial aspect of the test samples.

The samples were all stored in artificial saliva during the period of the experiment. The bleaching agents were applied to the labial aspect of the test specimens to a thickness of 1-2 mm (Figures 5A, 5B).

Group I received 35% hydrogen peroxide (Figure 5A) and Group II received 40% hydrogen peroxide (Figure 5B). Thirty-five percent hydrogen peroxide is available in a powder-liquid form when mixed turns into a turquoise-colored gel. It can be further light activated using blue phase light-emitting diode (LED) curing units. Forty percent hydrogen peroxide was chemically activated using a pH conditioner. It is available as a double-barreled syringe which when mixed produces a red color gel. A simple bleaching protocol was used in the study to bleach the test specimens (Table 1).

**Table 1:** The bleaching agent and bleaching protocol used for test samples in Group IA, Group IB, Group IIA, and Group IIB

Groups	Bleaching Agent	Bleaching Protocol
Group IA	35% hydrogen peroxide-based bleaching agent (Pola Office)	35% hydrogen peroxide-based bleaching agent of pH 6 applied for a total time of 10 minutes which included 3 minutes of light activation and 7 minutes without light activation.
Group IB	35% hydrogen peroxide-based bleaching agent (Pola Office)	35% hydrogen peroxide-based bleaching agents of pH 6 applied for a total of 30 minutes: three sessions of 10-minute application which included 3 minutes of light activation and 7 minutes without light activation.
Group IIA	40% hydrogen peroxide-based bleaching agent (Opalescence Boost)	Chemically activated 40% hydrogen peroxide-based bleaching agent of pH 8.5 applied for 10 minutes.
Group IIB	40% hydrogen peroxide-based bleaching agent (Opalescence Boost)	Chemically activated 40% hydrogen peroxide-based bleaching agent of pH 8.5 applied for a total of 30 minutes: three sessions of 10-minute applications.

Group IA: Test specimens received 35% hydrogen peroxide bleaching agent for a total time of 10 minutes which included three minutes of light activation and seven minutes without light activation. Group IB: Test specimens received 35% hydrogen peroxide bleaching agents for a total of 30 minutes, three sessions of 10-minute application, which included three minutes of light activation and seven minutes without light activation. Group IIA: Test specimens received chemically activated 40% hydrogen peroxide for 10 minutes. Group IIB: Test specimens treated with chemically activated 40% hydrogen peroxide for 30 minutes, three sessions of 10-minute applications.

**Figure 6:**
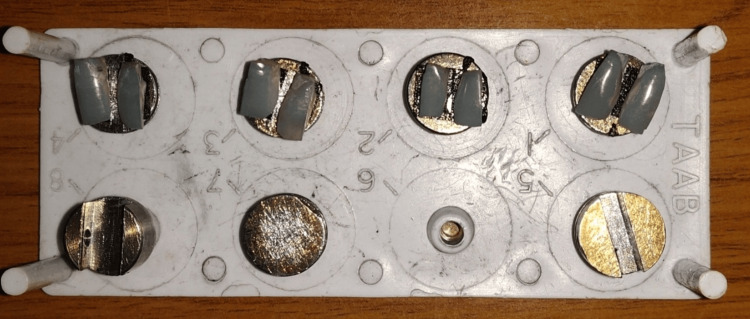
Samples in sputter coated in gold-palladium

All the control specimens of Group IA, Group IB, Group IIA, and Group IIB were not exposed to any bleaching agents. These specimens were stored in artificial saliva during the period of the experiment. Once the bleaching procedure was completed, the tooth specimens were washed under running water and blot-dried. Before examination with FESEM, the samples were placed in aluminum stubs and sputter coated in gold-palladium for 30 seconds (Figure 6).

**Figure 7:**
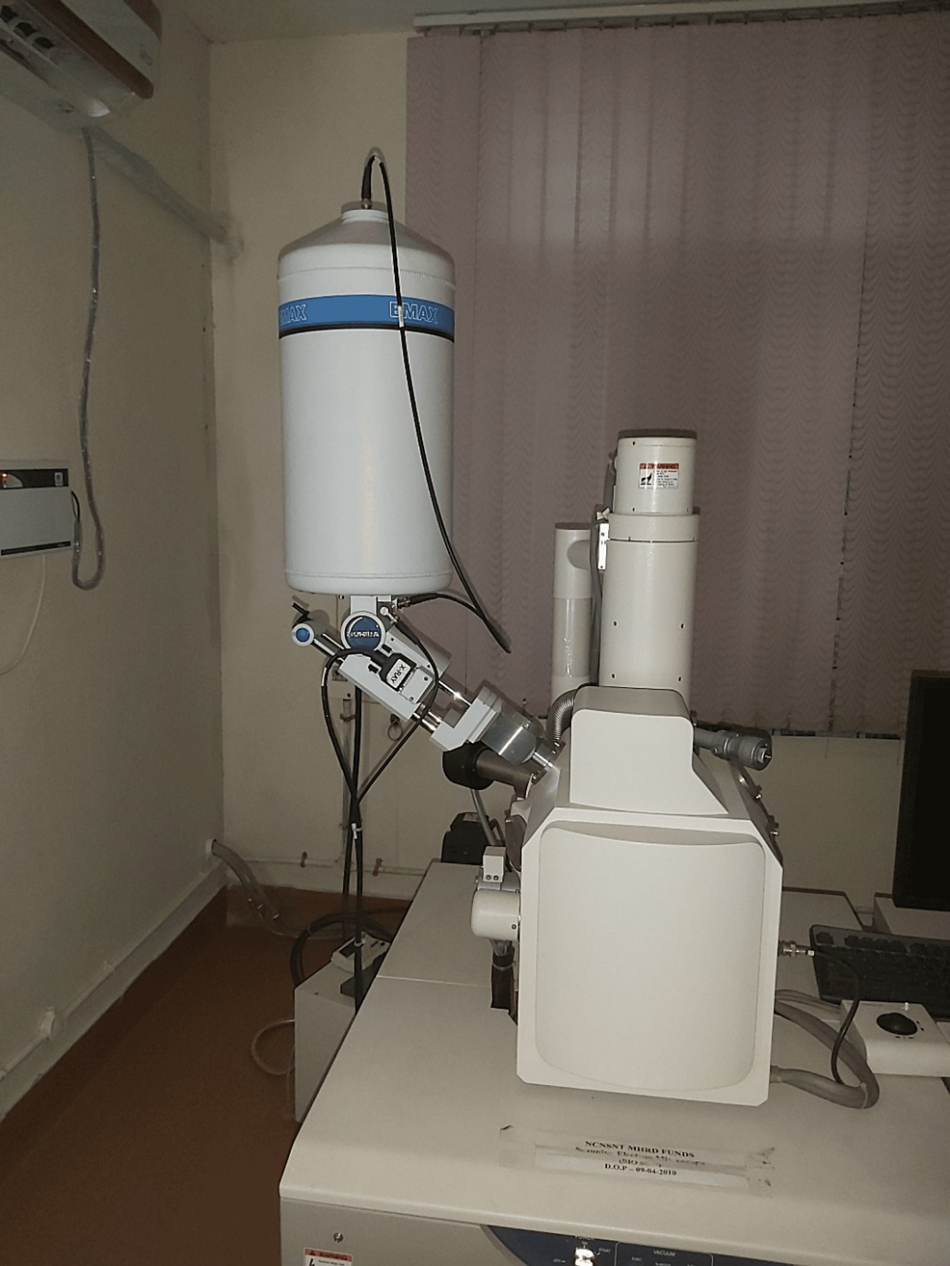
Field emission scanning electron microscope-energy dispersive spectroscopy unit

The tooth specimens were then mounted onto the FESEM holder. The middle third of the labial aspect of the enamel was adjusted to examine the topography and measure the concentration in weight % of the various elements on the enamel surface. A field emission scanning electron microscope (Hitachi N3400, Singapore) compatible with energy dispersive X-ray spectroscopy (Horiba Instruments, Japan) was utilized for the study (Figure 7).

The enamel was examined at 5,000× and 10,000× magnification with the FESEM using 2 kV under high vacuum mode and variable pressure. To measure the elemental composition of enamel before and after bleaching, EDS was utilized. The accelerating voltage was set at 10 kV with a working distance of 3.8 mm and resolution of 125.3 eV. The X-ray detector was set at 1-2 µm depth throughout the experiment under secondary electron (SE) mode at a magnification of 5,000×. The spectra and net intensity of the detected elements for each spot were collected at approximately 50 counts in seconds. Concentrations were determined after calculating the average percentage of the weight of a particular element at each spot. The EDS was used for line scans of the intensities of the Kx lines of oxygen, sodium, calcium, phosphorous, potassium, magnesium, and chlorine. The sample surface is irradiated with a high energy focused X-ray beam which leads to the emission of characteristic X-rays with wavelength specific to each element. Changes in the wavelength of the X-rays emitted indicate an alteration in the concentration of the elements analyzed in the study. The data obtained were entered into an Excel (Microsoft, Albuquerque, USA) spreadsheet and analyzed using Stata Statistics/Data Analysis special edition 12.0 (StataCorp LLC, College Station, USA). To describe the data descriptive statistics, mean and standard deviation were used. Paired t-test was used to find the significant difference between bivariate samples in independent groups. In all the above statistical tools, the probability value 0.05 is considered a significant level.

## Results

Results of the present study were subjected to statistical analysis to evaluate the chemical alterations in enamel following in-office dental bleaching. The results of FESEM analysis and EDS are listed below.

FESEM analysis

Control Group

**Figure 8:**
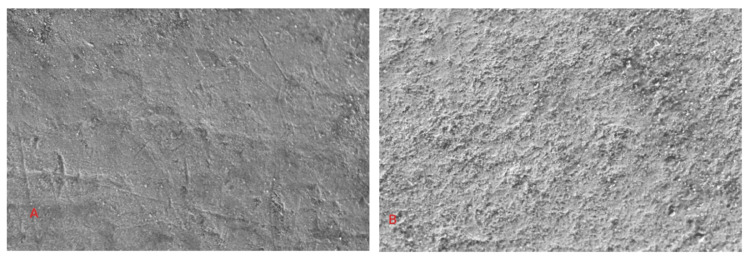
Representative FESEM image of control sample A: Representative FESEM image of control sample at 5,000× magnification. B: Representative FESEM image of control sample at 10,000× magnification. FESEM: field emission scanning electron microscopy.

Figures 8A, 8B represent the morphology of unbleached control samples.

The morphology of all enamel samples from all the control groups revealed similar surface features with few variations. The enamel surface showed ripple configurations representing perikymata grooves and imbrication lines, enamel rods, prismless enamel, a few scratches of various depths, a few particles scattered during the sample preparation, artifacts, and cracks. The surface is not completely smooth. The surface revealed the presence of very few scattered openings and surface irregularities.

Test Groups

**Figure 9:**
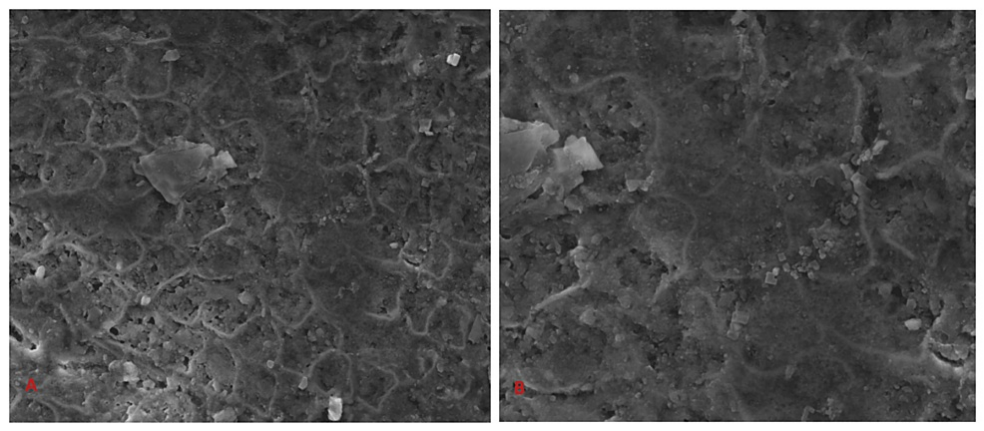
Representative FESEM image of test sample A: Representative FESEM image of test sample of Group IA at 5,000× magnification. B: Representative FESEM image of test sample of Group IIA at 10,000× magnification. FESEM: field emission scanning electron microscopy.

Figures 9A, 9B represent the bleached enamel surface of test samples under FESEM.

Enamel was deeply scored with many randomly oriented scratches, and exposed enamel prism was present. Rounded enamel rods with depressed rod boundaries were evident. Mild interprismatic dissolution is also evident. The perikymata and imbrication lines were shallower than the control group. Intermittent depressions of various diameters and depths were present. Widely spaced, randomly oriented scratches were also noticed. Scattered debris from the sample preparation littered the surface but had fewer particles when compared to the control groups. There is an increase in porosity, craters, and depressions with increased deposition of precipitates on the surface. The precipitates are mostly globular entities that measured around 1 µm in diameter. Their distribution on the enamel surface is in-homogenous. The precipitates can also be seen to agglomerate into larger masses on the enamel surface. Areas of erosion were evident in all groups.

EDS analysis

**Figure 10:**
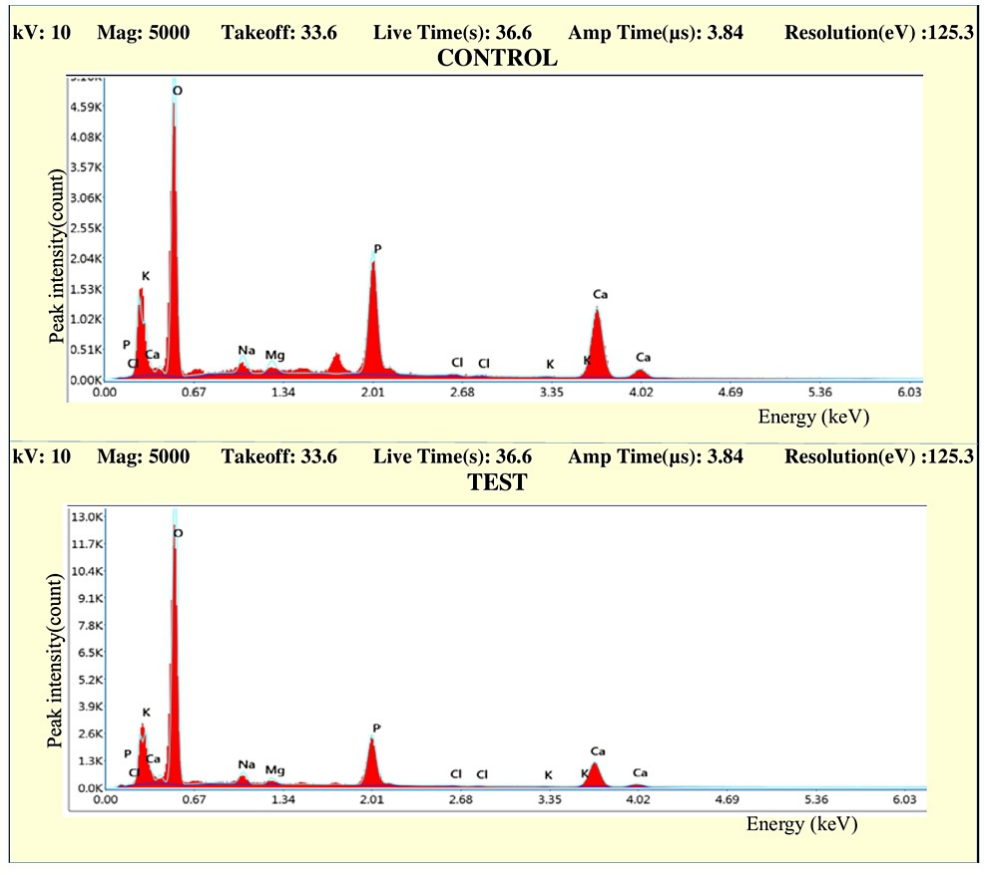
Graph comparing the concentration of elements (weight %) of control and test samples in Group IA

**Figure 11:**
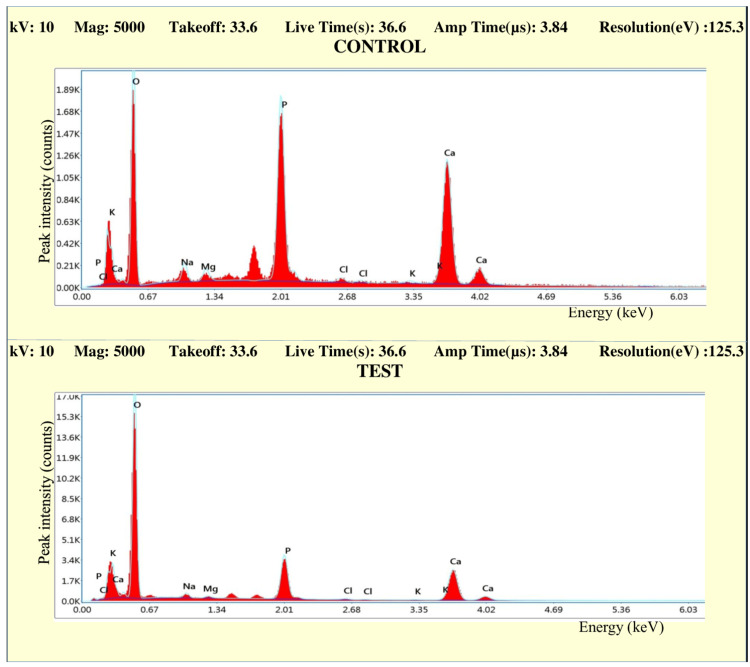
Graph comparing the concentration of elements (weight %) of control and test samples in Group IB

**Figure 12:**
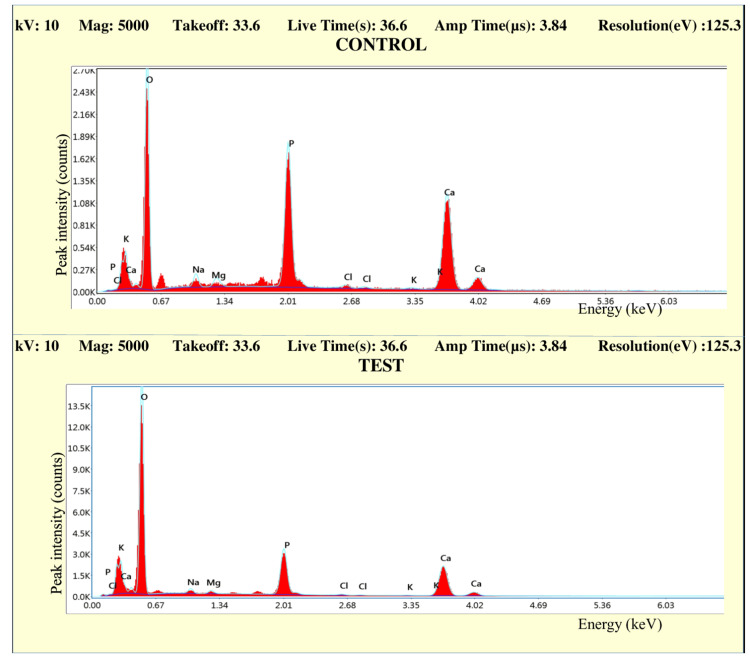
Graph comparing the concentration of elements (weight %) of control and test samples in Group IIA

**Figure 13:**
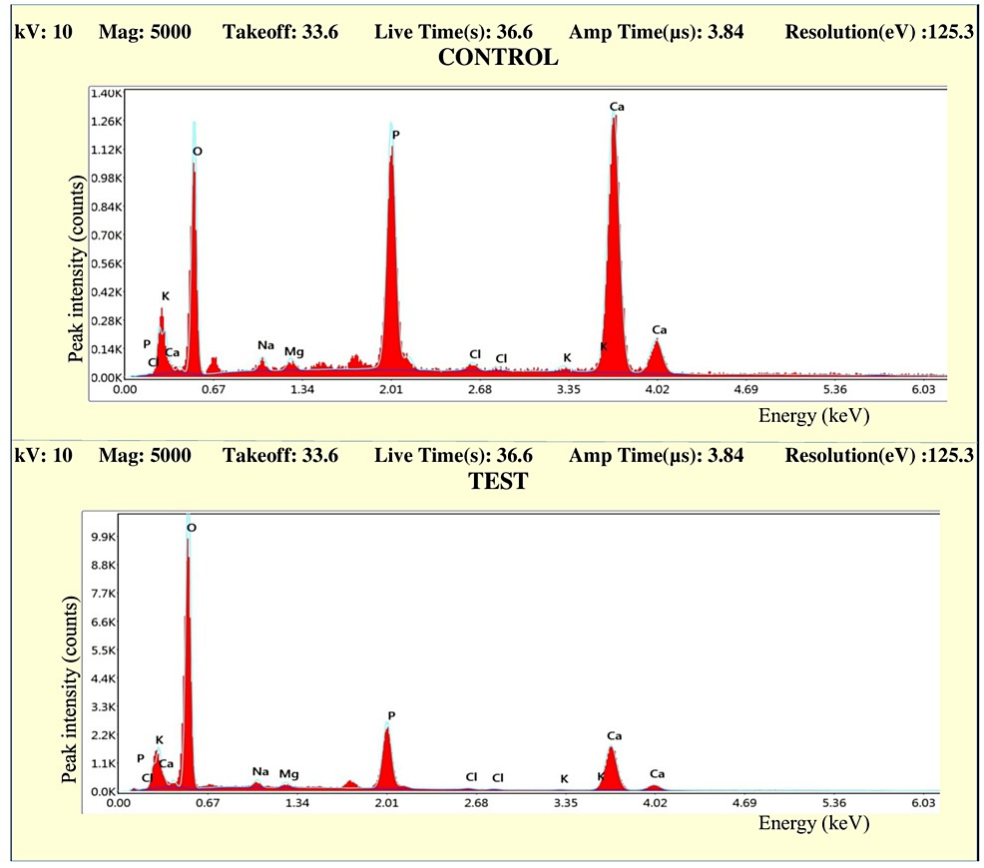
Graph comparing the concentration of elements (weight %) of control and test samples in Group IIB

The observations from the EDS analysis are made in the form of graphs. Figures 10-13 represent the comparison of the concentration of elements (weight %) between the control and test samples.

All the graphs of test groups display an observable reduction in the concentration (weight %) of minerals like calcium and phosphorous. The reduction in the concentration of other minerals like magnesium, chlorine, sodium, and potassium is observable but not significant. The increase in the peak of oxygen weight % can also be marked in all the graphs. The changes in the concentration of mineral density (weight %) in enamel were listed in the form of tables (Tables 2-7).

**Table 2:** Difference in the composition of enamel (weight %) between the control and test samples for different treatment times of 35% hydrogen peroxide The data is in mean ± SD format.

35% Hydrogen peroxide	Group IA (10 minutes )			Group IB (30 minutes)		
Elements	Control	Test	p value	Control	Test	p value
Oxygen	53.186 ± 5.7	50.446 ± 4.1	0.4096	36.16 ± 6.5	49.558 ± 4.4	0.0050
Sodium	2.136 ± 0.2	1.658 ± 0 .1	0.0028	1.63 ± 0.3	1.42 ± 0.3	0.3005
Magnesium	1.008 ± 0.11	0.688 ± 0.1	0.0008	0.864 ± 0.13	0.5 ± 0.15	0.0040
Phosphorous	16.154 ± 1.6	14.514 ± 1.0	0.0873	19.696 ± 1.5	15.292 ±1.6	0.0021
Chlorine	0.402 ± 0.1	0.422 ± 0.1	0.8433	0 .678 ± 0.1	0.486 ± 0 .1	0.0061
Potassium	0 .27 ± 0.1	0.264 ± 0.03	0.9045	0.55 ± 0.12	0.192 ± 0.02	0.0002
Calcium	34.3 ± 2.3	31.372 ± 1.8	0.0567	40.426 ± 5.3	30.822 ± 0.8	0.0040

**Table 3:** Difference in the composition of enamel (weight %) between the control and test samples for different treatment times of 40% hydrogen peroxide The data is in mean ± SD format.

40% Hydrogen peroxide	Group IIA (10 minutes)			Group IIB (30 minutes)		
Elements	Control	Test	p value	Control	Test	p value
Oxygen	46.464 ± 3.3	50.728 ± 1	0.0248	35.192 ± 10	51.706 ± 1.7	0.0063
Sodium	1.518 ± 0.14	1.312 ± 0.2	0.0876	1.224 ± 0.3	1.228 ± 0.12	0.9773
Magnesium	0 .822 ± 0.03	0 .522 ± 0.03	0.0000	0.672 ± 0.12	0.434 ± 0.4	0.0036
Phosphorous	15.98 ± 1	13.054 ± 1.1	0.0022	18.268 ± 2	14.286 ± 0.5	0.0020
Chlorine	0.51 ± 0.1	0.49 ± 0 .1	0.7588	0.638 ± 0.5	0.444 ± 0.02	0.0057
Potassium	0.27 ± 0.04	0.288 ± 0.10	0.7404	0 .388 ± 0.22	0.172 ± 0.02	0.0663
Calcium	33.838 ± 2	30.496 ± 1.2	0.0060	43.618 ± 1	29.774 ± 0.1	0.0120

**Table 4:** Difference in the composition of enamel (weight %) between different treatment times when bleached with 35% hydrogen peroxide The data is in mean ± SD format.

Elements	10 minutes	30 minutes	p value
Oxygen	50.446 ± 4.12	49.558 ± 4.4	0.7498
Sodium	1.658 ± 0.14	1.42 ± 0.3	0.1402
Magnesium	0.688 ± 0.1	0.498 ± 0.15	0.0346
Phosphorous	14.514 ± 1.1	15.292 ± 1.6	0.3880
Chlorine	0.422 ± 0.2	0.486 ± 0.1	0.4951
Potassium	0.264 ± 0.1	0.192 ± 0.024	0.0972
Calcium	31.372 ± 1	30.822 ± 1	0.5425

**Table 5:** Difference in the composition of enamel (weight %) between different treatment times when bleached with 40% hydrogen peroxide The data is in mean ± SD format.

Elements	10 minutes	30 minutes	p value
Oxygen	50.728 ± 1	51.706 ± 1.7	0.2842
Sodium	1.312 ± 0.12	1.228 ± 0.11	0.4241
Magnesium	0.522 ± 0.03	0.434 ± 0.03	0.0025
Phosphorous	13.054 ± 1.1	14.286 ± 0.5	0.0624
Chlorine	0.49 ± 0.11	0.444 ± 0.02	0.3832
Potassium	0.288 ± 0.11	0.172 ± 0.02	0.0471
Calcium	30.696 ± 1.3	29.974± 1	0.3261

**Table 6:** Difference in the composition of enamel (weight %) between different concentrations of hydrogen peroxide at 10 minutes of exposure H_2_O_2_: hydrogen peroxide. The data is in mean ± SD format.

Elements	H_2_O_2 _35%	H_2_O_2_ 40%	p value
Oxygen	50.446 ± 4.1	50.728 ± 1	0.8852
Sodium	1.658 ± 0.14	1.312 ± 0.18	0.0113
Magnesium	0.688 ± 0.1	0. 522 ± 0.03	0.0019
Phosphorous	14.514 ± 1	13.054 ± 0.5	0.0691
Chlorine	0.422 ± 0.2	0.49 ± 0.108	0.5061
Potassium	0.264 ± 0.1	0.288 ± 0.1	0.7031
Calcium	31.372 ± 1.8	30.496 ± 1.2	0.3864

**Table 7:** Difference in the composition of enamel (weight %) between different concentrations of hydrogen peroxide at 30 minutes of exposure H_2_O_2_: hydrogen peroxide. The data is in mean ± SD format.

Elements	H_2_O_2_ 35%	H_2_O_2_ 40%	p value
Oxygen	49.558 ± 4.4	51.706 ± 1.64	0.3347
Sodium	1.42 ± 0.3	1.228 ± 0.12	0.2108
Magnesium	0.5 ± 0.15	0.434 ± 0.03	0.3690
Phosphorous	15.292 ± 1.6	14.286 ± 0.5	0.2182
Chlorine	0.486 ± 0.64	0.444 ± 0.25	0.2127
Potassium	0.192 ± 0.02	0.172 ± 0.02	0.2219
Calcium	30.822 ± 0.4	29.774± 1	0.0995

## Discussion

The present study was undertaken to evaluate the various deleterious morphological and elemental changes in enamel caused by the application of high concentrations of hydrogen peroxide with different pH values. FESEM-EDS was utilized for the investigation of enamel surface for morphological alterations and elemental changes. Under FESEM analysis, the test groups evidenced areas of erosion on the enamel surface. However, the FESEM images of different test samples showed that there were not many differences in enamel morphology with the use of different bleaching agents or bleaching protocols. The increase in the density of surface pitting can be attributed to the partial removal of the superficial enamel layer which includes organic precipitates, organic matrix, and surface minerals. The superficial erosive alteration is characterized as ‘peeling effect’ caused by the loss of the aprismatic layer which in turn exposes the enamel prism [11]. A frosty appearance of the enamel following bleaching is caused due to the deposition of a white precipitate on the surface [8]. Peroxides also affect the inter-prismatic and intra-prismatic areas of enamel leading to minimal deproteinization [12]. Damage to the enamel surface is also evidenced by the prominent perikymata, eroded surfaces, and shearing of the enamel rods [13]. The FESEM images of test samples obtained in this study are comparable to the Silverstone Type III pattern as there is an involvement of the peripheral portion of the crystals within the prisms, discontinuity of interprismatic enamel, and the irregular surface [14]. The FESEM findings in the present study are following the observations made by Titley et al. [8], Miranda et al. [15], and McGuckin and Babin [16].

The difference in the concentration of each element (weight %) such as oxygen, sodium, magnesium, phosphorous, chlorine, potassium, and calcium between control and test samples of Group IA and Group IB was compared, and results are made in Tables 2. In Group IA, there was a reduction in weight % of all the elements between the control and test samples. However, the difference was statistically significant only for sodium and magnesium. In group IB, a similar reduction in the weight % of the elements was noticed. The reduction was statistically significant for all elements except for sodium. The statistically significant reduction in mean weight % of magnesium in test samples of Group IA and Group IB indicates that the demineralization process occurred in the test samples following the application of hydrogen peroxide-based bleaching agents. The results are in accordance with the findings of Cakir et al., Coceska et al., and Vilhena et al. [10,13,14]. The difference in the concentration of each element (weight %) such as oxygen, sodium, magnesium, phosphorous, chlorine, potassium, and calcium between control and test samples of Group IIA and Group IIB was compared, and results are made in Tables 3. The decrease in the concentration of oxygen, magnesium, phosphorous, and calcium between the control and test samples is statistically significant in Group IIA, whereas in Group IIB, the reduction in concentration of minerals like oxygen, magnesium, phosphorous, chlorine, and calcium between the control and test samples is statistically significant. These results are in accordance with the findings of Cakir et al., Coceska et al., Vilhena et al., McCracken and Haywood, Goo et al., and Lee et al. [10,13,14,17-19]. Hence from the results recorded in Tables 2 and 3, it can be concluded that demineralization of enamel took place in the test samples post-bleaching with hydrogen peroxide irrespective of its concentration, pH, or treatment time. Tables 4, 5 represent the effect of bleaching on the weight % of elements of enamel when the test samples were treated with hydrogen peroxide of the same concentration and pH but for different time period. Table 4 shows changes in concentration (weight %) of elements when treated with 35% hydrogen peroxide of pH 6 for varying treatment times (10 minutes and 30 minutes). There was a decrease in the mean concentration (weight %) of elements such as oxygen, sodium, magnesium, potassium, and calcium with an increase in the treatment time. However, these results were statistically insignificant for all elements except magnesium. The statistically significant reduction in magnesium between the test samples of Group IA and test samples of Group IB highlights the higher level of demineralization with the increase in treatment times. Similar results were obtained when samples received 40% hydrogen peroxide of pH 8.5 for different treatment times (10 minutes and 30 minutes). The results show a decrease in the mean concentration (weight %) of elements like sodium, magnesium, potassium, chlorine, and calcium (Table 5). Except for the reduction in magnesium and potassium, all other minerals showed a statistically insignificant reduction in their concentration with the increase in treatment time. These results are as per the findings of Tezel et al. and Cakir et al. [10,20]. The effect of different concentrations and pH of hydrogen peroxide-based bleaching agents on the composition of enamel when applied for the same treatment time was analyzed and compared in Tables 6, 7. Here, the concentration and pH of the bleaching agent were kept as variables, while treatment time was kept as a constant. Table 6 represents a statistically significant reduction in weight % of magnesium and sodium when test samples were bleached for 10 minutes with different concentrations and pH of hydrogen peroxide. The results tabulated in Table 7 show no statistically significant reduction in the mean concentration (weight %) of any elements. From the above comparison, it can be concluded that the mineral density of enamel did not alter significantly with changes in the concentration and pH of the bleaching agent. This result is in accordance with results obtained by Pinto et al., Al-Salehi et al., and Xu et al. [21-23].

Hydrogen peroxide is an oxidative agent; the interaction between peroxide and the mineralized enamel causes an increase in the oxygen weight percentage [14]. A similar increase in the oxygen weight percentage is also noted in the present study. However, this increase in oxygen level is disadvantageous as it interferes with polymerization and hence compromises the adhesive properties of the interface between the enamel and restorative material [24]. A statistically significant loss in the calcium and phosphorous between the test and control groups evaluated in the study is attributed to the dissolution of hydroxyapatite crystals from enamel when exposed to peroxide [10,17,25,26]. Calcium and phosphorous are the main components of hydroxyapatite crystals, and their loss indicates irreversible alterations in enamel. The loss of magnesium indicates a demineralization process. The change in the magnesium contents is caused due to the evaporation of water on the enamel surface. Magnesium is the first element to be lost during a demineralization process and hence is considered the gold standard [10,13,14]. The study also showed a statistically significant reduction in the level of sodium, potassium, and chlorine. The reduction in their value can be attributed to changes in the organic portion of the enamel [27].

Even though EDS is an effective tool for elemental analysis, its inability to measure the concentration of elements with low atomic number especially when a thick sample is used should be considered as a drawback. Moreover, the exact intraoral environment that affects demineralization and remineralization processes cannot be replicated in an in vitro setup. Therefore, within the limitations of the study, it can be concluded that there is a significant alteration in the morphology and reduction in concentration (weight %) of elements like sodium, phosphorous, chlorine, potassium, and calcium on the application of a hydrogen peroxide-based bleaching agent irrespective of its concentration, pH, and treatment time. The statistically significant reduction in the concentration of magnesium on bleaching should be highlighted. The reduction in magnesium with an increase in the concentration and treatment time denotes that the amount of demineralization taking place is large, and the bleaching agents should be used with caution. The study also concludes that there was no relevant alteration in the morphology or chemical composition of enamel on applying a bleaching agent either with neutral or alkaline pH.

## Conclusions

The results obtained in the study highlight the erosive action of hydrogen peroxide-based bleaching agents. The study evidences the loss of minerals from enamel following bleaching. This mineral loss correlates with the concentration, pH of the bleaching agent, treatment time, and light or chemical activation. From the study, it can be concluded that bleaching agents containing high concentrations of hydrogen peroxide with acidic pH can cause mineral loss and surface erosion of enamel which is extremely detrimental to the tooth integrity. The study also provides a message to the clinicians performing in-office vital tooth bleaching that a lower concentration of hydrogen peroxide with an alkaline pH activated chemically or by light when used for the appropriate time period can yield good outcomes while causing less damage to the enamel.
